# Supplementary material for: Genomic Characterization of Marine Staphylococcus shinii Strain SC-M1C: Potential Genetic Adaptations and Ecological Role
Source: Microorganisms. 2025 Aug 9;13(8):1866. doi: 10.3390/microorganisms13081866 (PMC12388102; doi:10.3390/microorganisms13081866)
Supplement: Supplementary file 1 [file microorganisms-13-01866-s001.zip › microorganisms-3759062-supplementary.pdf]

Supplementary Material

Genomic Characterization of Marine *Staphylococcus shinii* Strain SC-M1C: Potential Genetic Adaptations and Ecological Role

Manar El Samak [1], Hasnaa Lotfy [2], Abdelrahman M. Sedeek [3], Yehia S. Mohamed [4,5,*] and Samar M. Solyman [1,2,*]

1 Department of Microbiology & Immunology, Faculty of Pharmacy, Suez Canal University, Ismailia 41522, Egypt

2 Department of Microbiology & Immunology, Faculty of Pharmacy, Sinai University—Elkantara Branches, Ismailia 41522, Egypt

3 Department of Microbiology & Immunology, Faculty of Pharmacy, Galala University, New Galala City, Suez 43511, Egypt

4 Department of Pathological Sciences, College of Medicine, Ajman University, Ajman P.O. Box 346, United Arab Emirates

5 Department of Microbiology and Immunology, Faculty of Pharmacy (Boys), Al-Azhar University, Cairo 11562, Egypt

* Correspondence: y.mohamed@ajman.ac.ae (Y.S.M.); samar.solyman@pharm.suez.edu.eg (S.M.S.)

Table S1. A summary of the genomic islands (GIs) identified by the IslandViewer 4 server within the *Staphylococcus shinii* strain SC-M1C genome

Island	Size (bp)		Mobility Elements	Key Genes	Predicted Functions
**I**	6,458	12	Transposase (IS4 family)	• Late competence protein (ComC)<br>• Rod shape-determining proteins (MreC/MreD)<br>• LSU ribosomal proteins L21p and L27p	• DNA uptake<br>• Cell morphology<br>• Stress tolerance
**II**	12,540	13	Mobile element proteins	• Poly-glycerol-phosphate α-glucosyltransferase	• Cell surface modification<br>• Structural adaptation
**III**	10,064	11	Transposase (IS200 family)	• Staphylococcal nuclease (SNase)<br>• Two-component system regulator (LuxR family)	• Stress response<br>• Nutrient transport regulation
**IV**	7,334	8	None detected	• Ribosome recycling factor<br>• GTP-sensing repressor (CodY)<br>• ATP-dependent protease subunits (HslU/HslV)<br>• XerC recombinase	• Translation efficiency<br>• Metabolic regulation<br>• Genome maintenance
**V**	14,465	14	None detected	• Duplicated ATPase component BL0693<br>• N-acetylmuramoyl-L-alanine amidase<br>• Teichoic acid biosynthesis protein C	• Nutrient uptake via ECF transporters<br>• Cell wall remodeling and degradation<br>• Ion homeostasis
**VI**	23,932	42	Phage-related genes	• Phage structural proteins (capsid, tail, portal)<br>• Phage integrase/repressor	• Prophage integration<br>• Lysogenic conversion
**VII**	20,611	33	Transposase (IS4 family); Phage terminase	• Methionine ABC transporter system<br>• Thioredoxin/glutathione-dependent thiol reductase<br>• Organic hydroperoxide resistance protein<br>• Citrate/H^+ symporter<br>• 3-dehydroquinate dehydratase	• Nutrient acquisition<br>• Oxidative stress resistance<br>• Detoxification
**VIII**	9,404	10	Mobile element protein	• Acetylxylan esterase-related enzyme<br>• TesB-like acyl-CoA thioesterase	• Carbohydrate degradation<br>• Lipid metabolism

**IX**	5,152	9	Phage terminase	• Glutamate-aspartate carrier protein	• Amino acid transport<br>• Phage-related integration
**X**	47,011	51	Cassette chromosome recombinase B; Transposon Tn554	• Branched-chain amino acid permease<br>• Homoserine O-acetyltransferase<br>• Arsenical/cadmium resistance operon<br>• Two-component system (YycFG)<br>• Abortive phage resistance protein<br>• Cell wall-anchored protein SasC	• Nutrient uptake<br>• Heavy metal resistance<br>• Stress response<br>• Phage defense
**XI**	11,939	12	Mobilization proteins	• Cell wall-anchored protein SasA<br>• Cadmium efflux system accessory protein<br>• Cadmium resistance protein<br>• Replication proteins	• Cell wall anchoring<br>• Cadmium resistance<br>• Plasmid replication
**XII**	41,890	48	Resolvase/integrase; Mobilization proteins	• Inner membrane protein translocase YidC<br>• mRNA interferase RelE<br>• YefM antitoxin<br>• Lysine decarboxylase family protein<br>• MFS-type transporter<br>• Replication proteins	• Membrane transport<br>• Toxin-antitoxin system<br>• Lysine metabolism<br>• Plasmid replication
**XIII**	83,760	126	Transposase; Mobile element proteins	• N-acetylmuramoyl-L-alanine amidase<br>• Predicted cell wall-anchored protein SasF<br>• Protein-disulfide isomerase (DsbA-related)<br>• Rhomboid family protein<br>• DNA translocase FtsK<br>• Putative UV-damage repair protein UvrX	• Cell wall remodeling<br>• Protein folding<br>• DNA repair<br>• Transposition<br>• Stress response
